# Optimising total RNA quality and quantity by phenol-chloroform extraction method from human visceral adipose tissue: A standardisation study

**DOI:** 10.1016/j.mex.2020.101113

**Published:** 2020-10-20

**Authors:** Dipayan Roy, Sojit Tomo, Anupama Modi, Purvi Purohit, Praveen Sharma

**Affiliations:** Department of Biochemistry, All India Institute of Medical Sciences (AIIMS), Jodhpur, Rajasthan, India

**Keywords:** RNA extraction, RNA purity, TRizol method, Visceral adipose tissue, Optimization

## Abstract

A multitude of challenges is faced during RNA extraction from human visceral adipose tissue (VAT) due to its atypical nature and a dearth of existing literature. Our study provides a convenient and inexpensive manual method using TRIzol reagent for the reproducible recovery of intact RNA from sparse human VAT samples. Fifty-two (52) samples were grouped and tested for the effect of different factors viz. initial VAT amount, TRIzol volume per unit tissue mass, residual fat following homogenisation and first centrifugation, an additional chloroform wash, and an additional ethanol wash on the extraction process. We found that increasing initial tissue mass and decreasing TRIzol volume simultaneously improved RNA yield and purity. A fat layer removal step and additional ethanol wash further propel the A260/280 and A260/230 to their desired values. Our modifications in the isolation protocol were combined and tested through reverse transcriptase quantitative PCR, which yielded consistent results, upholding our optimisation.

## Graphical abstract

**Figure:**
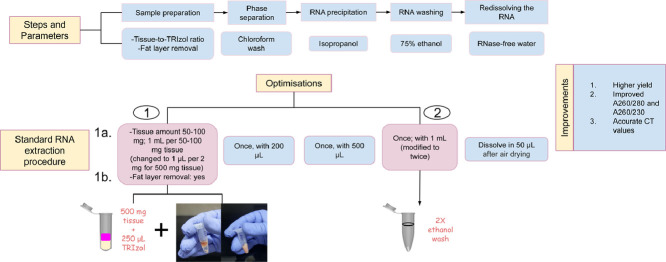

Specifications table

**Table:** 

Subject Area	Biochemistry, Genetics and Molecular Biology
More specific subject area	RNA extraction and quantification
Method name	An optimised method for RNA extraction from human visceral adipose tissue using TRIzol reagent
Reagents/tools	Sterile tissue-collection container Petri dish Scalpel and sterile surgical blades; forceps Tissue homogeniser Analytical balance 1.5 mL and 2 mL capped tubes Micropipettes and pipette tips Microcentrifuge 1% phosphate buffer saline (PBS) TRIzol reagent Chloroform Isopropyl alcohol 75% ethyl alcohol [prepared by 3:4 dilution from absolute alcohol] Diethyl pyrocarbonate (DEPC)-treated water Heating plate
Experimental design	We used TRIzol (HiMedia RNA-XPress™ Reagent), a commercially available RNA extraction reagent. The manufacturer's protocol for the reagent is designed after the single-step acid-phenol-chloroform RNA extraction method first described by Chomczynski and Sacchi in their seminal 1987 paper [1. Chomczynski P, Sacchi N. Single-step method of RNA isolation by acid guanidinium thiocyanate-phenol-chloroform extraction. Anal Biochem 1987; 162: 156–159.], which they further updated in 2006 [2. Chomczynski, P., & Sacchi, N. (2006). The single-step method of RNA isolation by acid guanidinium thiocyanate–phenol–chloroform extraction: twenty-something years on. Nature Protocols, 1(2), 581–585.]. Our optimised version includes modifications made upon these existing methodologies.
Trial registration	Not applicable
Ethics	This study conforms to the guidelines of the Institutional Ethics Committee (IEC), AIIMS, Jodhpur; written informed consent was signed from each participant before enrolling into the study.
Value of the Protocol	Our customised protocol uses a decreased TRIzol volume per unit mass of tissue which gives better quality RNA. Further, increasing the amount of tissue to 500 mg will improve the total RNA yield. We have added an additional step of ethanol wash to remove any residual impurities like salts or phenol and observed that it improves the RNA purity. We conclude that total RNA extraction by the phenol-chloroform method using TRIzol can be optimally utilised by increasing tissue amount, removing the fat layer, optimising the ratio of TRIzol to VAT, and including an extra ethanol wash, all of which improve the quality and purity of RNA.

## Description of protocol

### Background

Physicochemical idiosyncrasies of adipose tissue (AT) limit the efficacy of phenol-chloroform extraction protocols for total RNA isolation thereof. Larger cell size and high triglyceride content in mature adipocytes (the latter further increased in conditions like obesity) and low cell count give rise to various technical difficulties [3]. Furthermore, tissue samples received from operative procedures are scanty, cannot be processed immediately, and may contain other cells such as pre-adipocytes, endothelial cells, and fibroblasts [4,5]. A combination of these factors ultimately results into poor RNA yield, impurities (protein, carbohydrate, residual phenol or guanidine, salts), degradation and loss of small RNAs, and unacceptable purity (low A260/A280 and A260/A230 ratio), which further affect the accuracy and reliability of downstream applications [6], [7], [8]. AT-specific RNA isolation protocols are seldom available from suppliers or manuals. Moreover, although several groups throughout the years have worked on modifying and comparing existing protocols to improve the quality and integrity of isolated RNA [3,4,5,7,[9], [10], [11], [12], [13], [14], amongst others], there is a dearth of studies which give a comprehensive account of manual RNA isolation from human VAT.

### Experimental design

We aimed to provide a suitable, inexpensive method to retrieve total RNA from scanty human VAT samples by modifying the manual RNA isolation method through varying the amount of tissue, the volume of TRIzol reagent, and a step each for fat layer removal, and additional ethanol wash. The VAT samples were taken from fifty two (52) different patients, devoid of any complications or acute inflammatory conditions. Our exclusion criteria ruled out any patient having an existing condition that would affect the VAT architecture.

### Reagents/tools

• Sterile containers for the collection of tissue
• Petri-plates
• Scalpel and sterile surgical blades
• Forceps
• Gloves (preferably sterile)
• Analytical balance
• Tissue homogeniser
• 1.5 mL and 2 mL capped tubes
• Pipettes (100–1000 mL, 20–200 mL) and pipette tips
• Microcentrifuge
• 1% phosphate buffer saline (PBS) [prepared by 1:10 dilution from 10% stock solution and refrigerated at 4 °C]
• TRIzol reagent
• Chloroform
• Isopropyl alcohol
• 75% ethyl alcohol [prepared by 3:4 dilution from absolute alcohol]
• DEPC-treated water
• Heating plate
• Ice packs

### Pre-preparation

• Autoclave all glassware and surgical instruments (scalpel, scissor, forceps) before use. Pre-identify and label the microcentrifuge tubes.
• Immediately submerge the VAT samples, collected from freshly excised abdominal fat tissue, into the sterile container in 1% PBS and transfer to the laboratory where extraction will take place.
• Set the centrifuge to 4 °C well in advance.
• Clean the working bench properly with 70% ethyl alcohol or any other decontaminant.

### Protocol

(Optimisations and relevant justifications are mentioned in the statements marked with an *)

### Sample preparation and homogenisation

1. Wash the fresh tissue samples with 1% PBS solution and weigh it with the analytical balance.

* We suggest a maximum of 500 mg of pure VAT. However, excised adipose tissue sample mass varies widely depending on the surgery and availability of VAT in the patient. Hence, we suggest to take the maximum amount of tissue available (not more than 500 mg) and adjust the TRIzol volume accordingly.

2. Dissect the samples accurately to ensure pure adipose tissue and mince it into fine pieces to provide large enough surface area for digestion reagent. Carefully put the minced tissue in a 2 mL tube and homogenise with homogeniser.

We used a teflon homogenizer (GenetixBiotech), with a speed of 3000 rpm in a discontinuous manner, while allowing 5–10 s per stroke. The tubes were kept on ice while the process was being carried out.

* Use a 2 mL tube for 500 mg VAT as it will provide ample space required for the homogenisation of the high tissue volume. However, when working with a lesser mass of VAT, use a 1.5 mL tube, because it makes the pipetting during the fat layer separation much more accessible.

3. Centrifuge the homogenate at 12,000 x g for 10 min.

A fat layer will appear on the upper surface of the aqueous phase.

4. Carefully remove the fat layer by pipetting.

* The manufacturer's protocol suggests to transfer the aqueous supernatant to a separate tube and proceed with the subsequent steps. However, they have used 1 mL TRIzol in their protocol. With the lesser volume of TRIzol reagent we are using (less than or equal to 250 μL), separating the supernatant will decrease the yield further and not always practically possible.

### Phase separation

5. Incubate the homogenised sample at room temperature for 5 min.

6. Add 200 μL of chloroform; shake vigorously.

7. Incubate on ice for 15 min.

8. Centrifuge at 12,000 x g at 4 °C for 15 min.

After centrifugation, the mixture will separate into a lower dark pink organic phase (protein), whitish interphase (containing DNA), and an upper aqueous phase (containing RNA).

### RNA precipitation

9. Transfer the upper aqueous layer carefully to another 1.5 mL tube. Add 500 μL of isopropyl alcohol to it. Vortex and incubate the sample on ice for 20 min.

10. Centrifuge at 12,000 x g at 4 °C for 12 min.

A small, whitish pellet will be visible after this step. However, it may not be visible in case of less amount of tissue sample or low RNA concentrations.

### RNA wash

11. Discard the supernatant. Add 1 mL of 75% ethanol and vortex for a few seconds.

12. Centrifuge at 7,500 x g at 4 °C for 5 min.

Repeat the RNA washing steps 11 and 12. Some remaining ethanol after discarding the first supernatant is fine.

### RNA solubilisation

13. Discard the supernatant and air-dry the RNA pellet.

The tube can initially be kept upside down on a piece of clean tissue paper for a few minutes.

14. Add 50 μL DEPC-treated water to dissolve the RNA pellet.

The volume of water for dissolution of the pellet can vary. If the initial tissue amount is suboptimal and RNA pellet is not visible, then 30 μL water would suffice.

15. Heat the tubes on a heating plate at 60 °C for 10 min.

16. Quantify RNA concentration and purity with a microplate reader (BioTek). Store at −80 °C for downstream application later.

### Optimising VAT amount and TRIzol-to-tissue ratio

A glaring problem that arises while extracting RNA from adipose tissue is the less amount of initial tissue received. Moreover, the usual volume of TRIzol used (1 mL per 50–100 mg of tissue) according to the manufacturer's protocol, which yields a reasonably good amount of RNA for tissues like liver, spleen, kidney, placenta, skeletal muscle and brain, does not work equally well for VAT. The yield with the same amount of starting tissue is much lesser, with suboptimal 260/280 and 260/230 ratio. Specifically, 260/230 is most affected by larger TRIzol volumes.

**Fig. 1:**
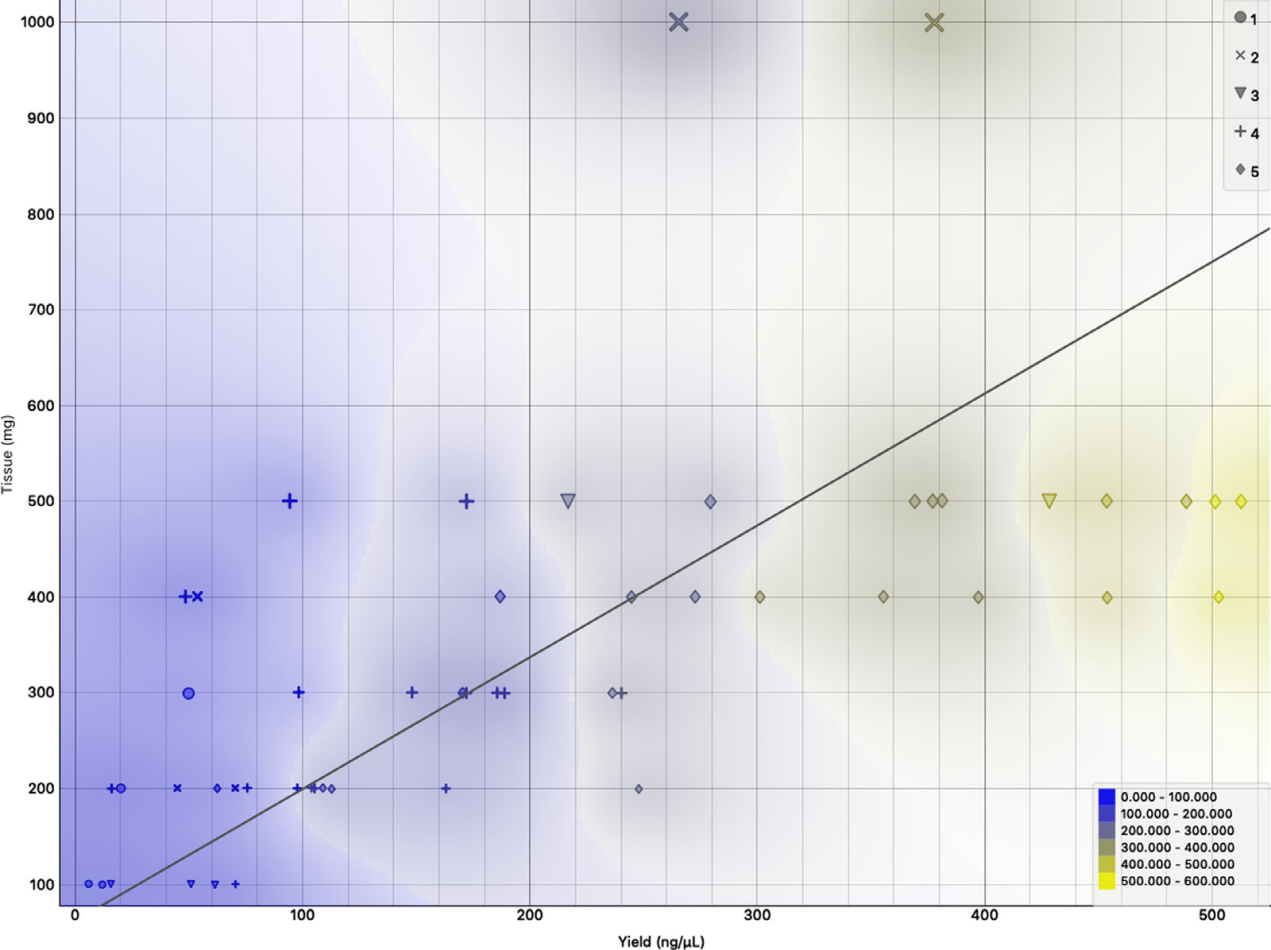
Effects on RNA concentration as a function of mass of adipose tissue used. RNA concentration showed a strong positive correlation with tissue mass (Spearman's rank correlation coefficient ρ=0.759, *p*<0.001). The points are categorised according to the volume of TRIzol (in μL) used per unit mass (in mg) of tissue.

To determine whether the initial amount of tissue taken affects the resulting RNA yield, we apportioned the freshly excised AT into six different variants, viz. 100 mg, 200 mg, 300 mg, 400 mg, 500 mg, and 1000 mg in weight, as measured by a digital analytical balance (Sartorius). The RNA yield showed a positively linear trend with increasing tissue amount (Spearman's rank correlation coefficient ρ = 0.76, *p*<0.001, Fig. 1).

Residual phenol from the extraction procedure is known to interfere with RNA purity. To optimise the TRIzol volume per unit AT mass, variations for each of the amounts mentioned above in five different ratios: 4:1, 2:1, 1:1, 2:3, and 1:2 were taken before being subjected to homogenisation. We observed a significant difference in 260/230 ratio between the groups while the intergroup difference in 260/280 ratio was not significant (Table 1). Descriptive analyses of the data further showed a steady increase of the 260/230 ratio as the TRIzol volume was reduced, being closest to its optimal for the fifth group. To see which groups differ from each other, we proceeded with a post-hoc analysis that revealed that using a 1:2 TRIzol-to-tissue ratio yields significantly better results in comparison to the other four groups individually.

**Table 1:** The comparison of RNA yield (ng/mg), A_260/280_ and A_260/230_ in samples extracted by different volumes of TRIzol reagent (expressed as median and Q1-Q3; Q1: 1st quartile, Q3: 3rd quartile).

Group	TRIzol volume (μL) per unit mass of tissue (mg)	No. of samples (*n*=)	Yield (ng/mg)*	260/280 ratio	260/230 ratio*
1	4:1	4	5.60 (4.56–6.71)	1.74 (1.65–1.86)	0.20 (0.16–0.52)
2	2:1	5	13.30 (11.20–17.60)	2.01 (1.69–2.04)	0.60 (0.29–1.03)
3	1:1	5	25.80 (21.65–30.95)	1.91 (1.77–2.04)	0.97 (0.96–1.27)
4	2:3	16	25.35 (17.04–31.06)	1.97 (1.92–2.02)	1.22 (1.16–1.68)
5	1:2	22	37.93 (28.99–49.50)^a^	1.95 (1.89–1.99)	1.79 (1.72–1.96)^b^

*p<0.001 (Kruskal-Wallis rank sum test).

Post-hoc analysis by pairwise comparisons using Wilcoxon rank sum test revealed:.

a adjusted *p*<0.05 with groups 1, 2 and 4.

b adjusted *p*<0.05 with all other groups.

Following the significant positive correlation of RNA yield with tissue amount and improved purity with decreasing TRIzol volumes, we rearranged the samples combining various tissue masses (200 mg, 300 mg, 400 mg, and 500 mg) with 2:3 and 1:2 TRIzol-to-tissue ratio. The combined groups (outlined in Table 2) were then compared for total RNA yield (ng/μL), total RNA yield (ng/mg of VAT), 260/280 ratio, and 260/230 ratio, and significant intergroup differences (*p*<0.001, *p* = 0.03, *p* = 0.013, and *p* = 0.003 respectively) were revealed for all four outcomes. The distribution showed a steady increase for yield across the groups while the 260/230 ratio was significantly improved in groups three, six, and eight compared to group one. Furthermore, the ng/mg RNA yield was significantly higher in group six compared to group one, and in group eight compared to groups one and seven, indicating that a lower tissue-to-TRIzol ratio of 1:2 indeed results in better quality and yield of RNA.

**Table 2:** The comparison of RNA yield (in both ng/μL and ng/mg), A_260/280_ and A_260/230_ in samples extracted using combination of different tissue amounts and TRIzol volumes (expressed as median and Q1-Q3).

Groups	Tissue mass (mg); TRIzol volume (μL)	*n*=	Yield (ng/μL)*	Yield (ng/mg) [according to tissue mass]	Yield (ng/mg)*	260/280 ratio*	260/230 ratio*
Group1	200; 2:3	6	101.03 (81.5–105.1)	26.19 (20.4–28.1)	25.28 (20.4–26.3)	1.97 (1.88–2.07)	1.20 (0.73–1.33)
Group2	200; 1:2	4	111.24 (97.6–147.0)		27.83 (24.4–36.8)	1.93 (1.87–1.97)	1.72 (1.55–1.77)
Group3	300; 2:3	6	178.72 (154.2–188.4)	29.79 (27.5–33.5)	29.78 (25.7–31.4)	1.98 (1.95–2.01)	1.72 (1.60–1.88)^i^
Group4	300; 1:2	2	203.49 (187.0–220.0)		33.93 (31.2–36.7)	2.02 (2.01–2.03)	1.84 (1.77–1.90)
Group5	400; 2:3	2	209.48 (189.9–229.1)	35.89 (30.7–48.4)	20.63 (13.4–27.9)	2.09 (2.05–2.11)	1.49 (1.28–1.69)
Group6	400; 1:2	8	328.37 (265.9–411.7)^a^		41.05 (33.2–51.5)^d^	1.88 (1.85–1.92)^g^	1.84 (1.77–2.11)^j^
Group7	500; 2:3	2	133.27 (113.8–152.8)^b^	37.94 (30.2–48.0)	13.33 (11.4–15.3)^e^	1.97 (1.95–1.98)	1.17 (1.16–1.18)^k^
Group8	500; 1:2	8	417.83 (375.2–492.1)^c^		41.78 (37.5–49.2)^f^	1.98 (1.96–1.99)^h^	1.84 (1.79–1.96)^l^

*p<0.05 (Kruskal-Wallis rank sum test).

The ng RNA per tissue is presented for each of the four groups of initial tissue amounts

Post-hoc analysis by pairwise comparisons using Wilcoxon rank sum test revealed significant differences (*p*<0.05) between:.

a RNA yield of Group6 vs. Group1 (*p* = 0.001), Group6 vs. Group2 (*p* = 0.016), and Group6 vs. Group3 (*p* = 0.003).

b RNA yield of Group7 vs. Group6 (*p* = 0.044).

c RNA yield of Group8 vs. Group1 (*p* = 0.001), Group8 vs. Group2 (*p* = 0.004), Group8 vs. Group3 (*p* = 0.001), Group8 vs. Group4 (*p* = 0.044), Group8 vs. Group7 (*p* = 0.044).

d Yield (ng/mg) of Group6 vs. Group1 (*p* = 0.016).

e Yield (ng/mg) of Group7 vs. Group6 (*p* = 0.014).

f Yield (ng/mg) of Group8 vs. Group1 (*p* = 0.008), Group8 vs. Group7 (*p* = 0.009).

g 260/280 ratio of Group6 vs. Group3 (*p* = 0.017), Group6 vs. Group4 (*p* = 0.049), and Group6 vs. Group5 (*p* = 0.049).

h 260/280 ratio of Group8 vs. Group6 (*p* = 0.003).

i 260/230 ratio of Group3 vs. Group1 (*p* = 0.041).

j 260/230 ratio of Group6 vs. Group1 (*p* = 0.006).

k 260/230 ratio of Group7 vs. Group6 (*p* = 0.049).

l 260/230 ratio of Group8 vs. Group1 (*p* = 0.001), Group8 vs. Group2 (*p* = 0.049), and Group8 vs. Group7 (*p* = 0.044).

We found a significant difference between RNA yield of group six compared to groups one, two, and three, while the yield for group eight was significantly different compared to groups one, two, three, four, and seven. The 260/280 ratio was higher in group eight compared to group six. For the 260/230 ratio, the differences observed between group eight and groups one, two, and seven were statistically significant.

### Fat layer removal step and additional ethanol wash for improving RNA purity

**Fig. 2:**
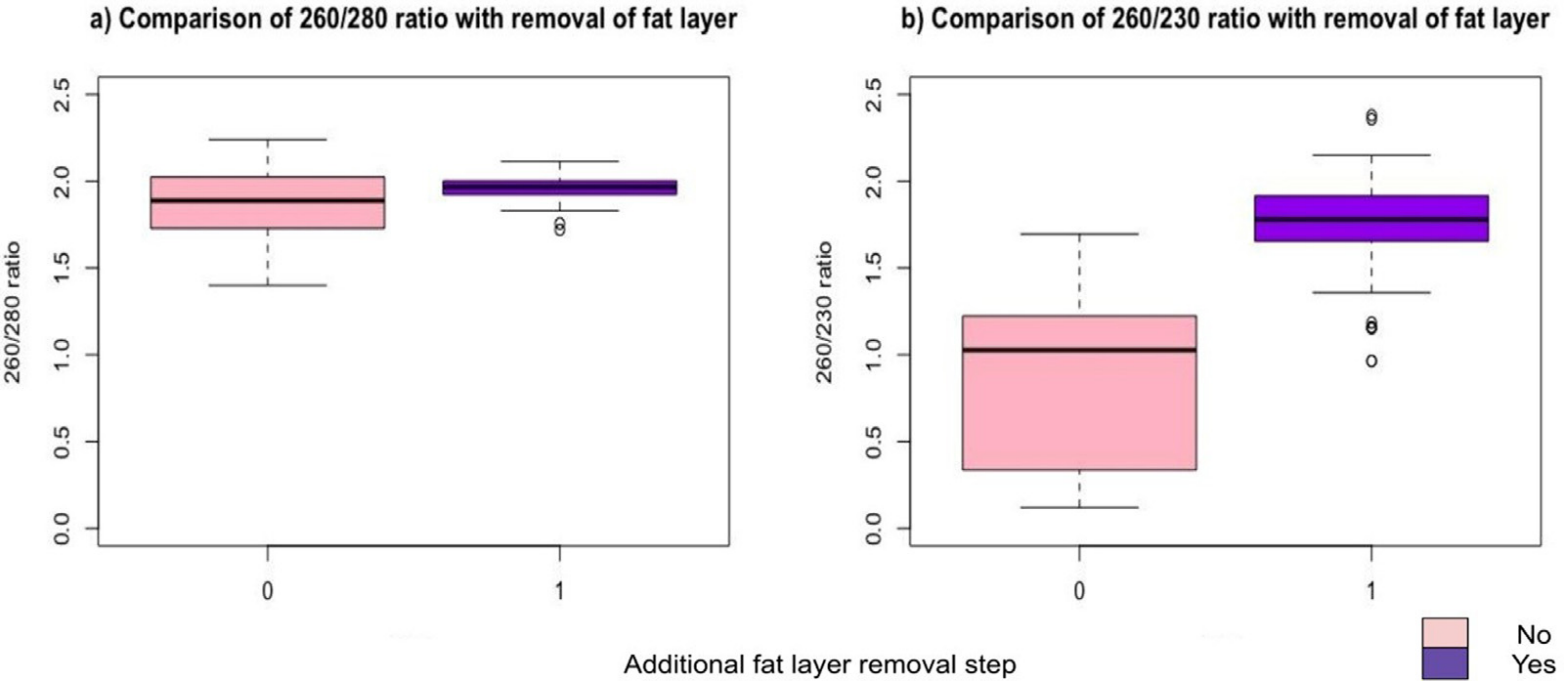
Effect of fat layer removal step. After the first centrifugation step, the fat layer was removed. Code 0=No additional step, 1=Additional step of fat removal. Boxplots show that a) 260/280 ratio undergoes no significant improvement (*p* = 0.14); b) 260/230 ratio has significant improvement with the additional step (*p*<0.001). * *p*<0.05 (Two sample Wilcoxon rank sum test with continuity correction).

We evaluated whether the residual fat after the first centrifugation step interferes with pure RNA extraction by comparing A_260/280_ and A_260/230_ in samples processed with or without the fat layer removal step. While A_260/280_ did not show any significant changes, A_260/230_ ratio showed a significant increase towards the desired value compared to those without this step (Median [Q1-Q3] 1.78 [1.66–1.92] compared to 1.03 [0.34–1.22], *p*<0.001, Fig. 2).

**Fig. 3:**
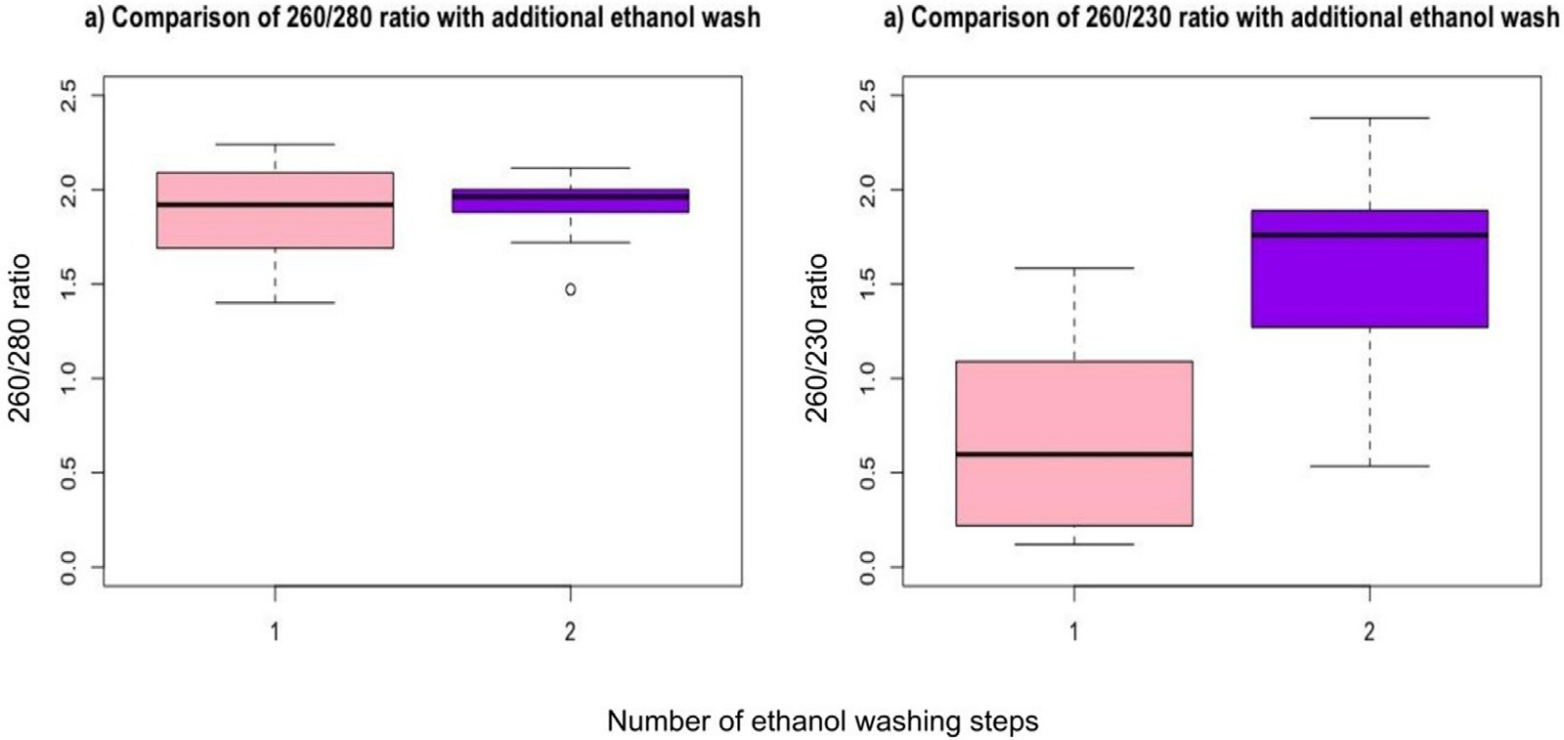
Effect of an additional ethanol wash after RNA precipitation. Code 1=single ethanol wash, 2=double ethanol wash. Boxplots show that a) 260/280 ratio undergoes no significant improvement (*p* = 0.76); b) 260/230 ratio has significant improvement with the additional step (*p*<0.001). * *p*<0.05 (Two sample Wilcoxon rank sum test with continuity correction).

We tried two ethanol washing steps instead of a single washing to remove any remaining impurities in the form of salts. We observed that the A_260/230_ ratio showed significant improvement with two washing steps compared to one (Median [Q1-Q3] 1.76 [1.29–1.88] compared to 0.60 [0.24–1.09], *p*<0.001, Fig. 3).

## Method validation

### Agarose gel electrophoresis

Agarose gel electrophoresis of the extracted RNA was carried out for selected samples on a native 2% agarose gel using TAE buffer with RiboRuler Low Range RNA Ladder (Thermo Fisher Scientific, Massachusetts, US) following the manufacturer's protocol.

### Reverse transcription

After maximising the RNA yield and purity through the modifications as mentioned earlier, we chose five samples for comparison between our optimised protocol and the existing protocol (Table 3). We selected representative samples from groups 6 and 8 (in table 2) as they had the highest RNA yield (μg/mg) with acceptable 260/280 and 260/230 ratios. The same tissues were also subjected to the existing manufacturer's protocol. In order to verify our findings, we had further subjected these five different samples to quantitative PCR. We used the miScript II RT Kit (QIAGEN) to yield complementary DNA (cDNA) from the samples. Template RNA was thawed on ice. Reagents were thawed at room temperature. Since we were carrying out quantification of both mature mRNA as well as miRNA, we used the 5x miScript HiFlex Buffer suited for the purpose. The RNA quantity in the reaction mixture was kept at 0.5 μg, following the recommendation of the manufacturer's protocol. The 10 μL of reaction mixture contained 2 μL 5x miScript HiFlex Buffer, 1 μL 10x miScript Nucleics Mix, 1 μL miScript Reverse Transcriptase Mix, and 6 μL of RNase-free water and template RNA. Reverse transcription was carried out at 37 °C for 60 min, and subsequently, 95 °C for 5 min to inactivate the reaction.

**Table 3:** Comparison of the existing protocol and our optimised protocol.

Steps and variations	Existing protocol	Optimised protocol
**Homogenisation and tissue lysis**		
Initial tissue amount (in mg)	50–100	500
Tissue-to-trizol ratio	10:1 [1 mL per 100 mg tissue]	1:2 (250 μL per 500 mg of tissue)
Fat layer removal	Centrifugation at 12,000 x g for 5 min at 4–10 °C and transfer of supernatant to a new tube	Centrifugation at 12,000 x g for 5 min at 4 °C and upper fat layer carefully pipetted out
Chloroform wash	Once; 200 μL per 1 mL of TRIzol	Once; 200 μL
**RNA precipitation**		
Isopropanol wash	Transfer aqueous phase to a new tube and add 0.5 mL of isopropanol	Transfer aqueous phase to a new tube and add 0.5 mL of isopropanol
	Incubate and centrifuge; discard the supernatant	Incubate and centrifuge; discard the supernatant
**RNA wash by 75% ethanol**		
Ethanol wash	Discard supernatant, add 75% ethanol, vortex and centrifuge at 7500 x g for 5 min at 4 °C	Discard supernatant, add 75% ethanol, vortex and centrifuge at 7500 x g for 5 min at 4 °C
	–	Repeat the above step one more time
Air dry of RNA pellet	5–10 min	5–10 min
**RNA solubilization**		
Resuspension of pellet	20–50 μL of RNase-free water	50 μL of RNase-free water
Heat incubation	50–60 °C for 10 min	50–60 °C for 10 min

### Quantification by real-time PCR

cDNA was amplified using miScript^Ⓡ^ SYBR^Ⓡ^ Green PCR Kit (QIAGEN, Hilden, Germany) on a BioRad CFX96 Real-Time system. Each 10 μL PCR reaction contained 5 μL 2x QuantiTect SYBR Green PCR Master Mix, 1 μL 10x miScript Universal Primer, 1 μL 10x miScript Primer Assay, 2 μL of RNase-free water and 1 μL of template cDNA, keeping in mind that the final concentration of cDNA was 3 ng per reaction. The reactions were then incubated at 95 °C for 15 min to activate the HotStarTaq DNA Polymerase. It was followed by 40 cycles of 94 °C for 15 s, 55 °C for 30 s, and 70 °C for 30 s. We assayed for SMAD7 (Mothers against decapentaplegic homologue 7), MIR181 (microRNA-181) and two housekeeping genes GAPDH (glyceraldehyde-3-phosphate dehydrogenase) and RNU6 (U6 small nuclear RNA) in five VAT samples to compare their cycle thresholds (Ct) values. All samples were run in duplicates.

## Results

**Fig. 4:**
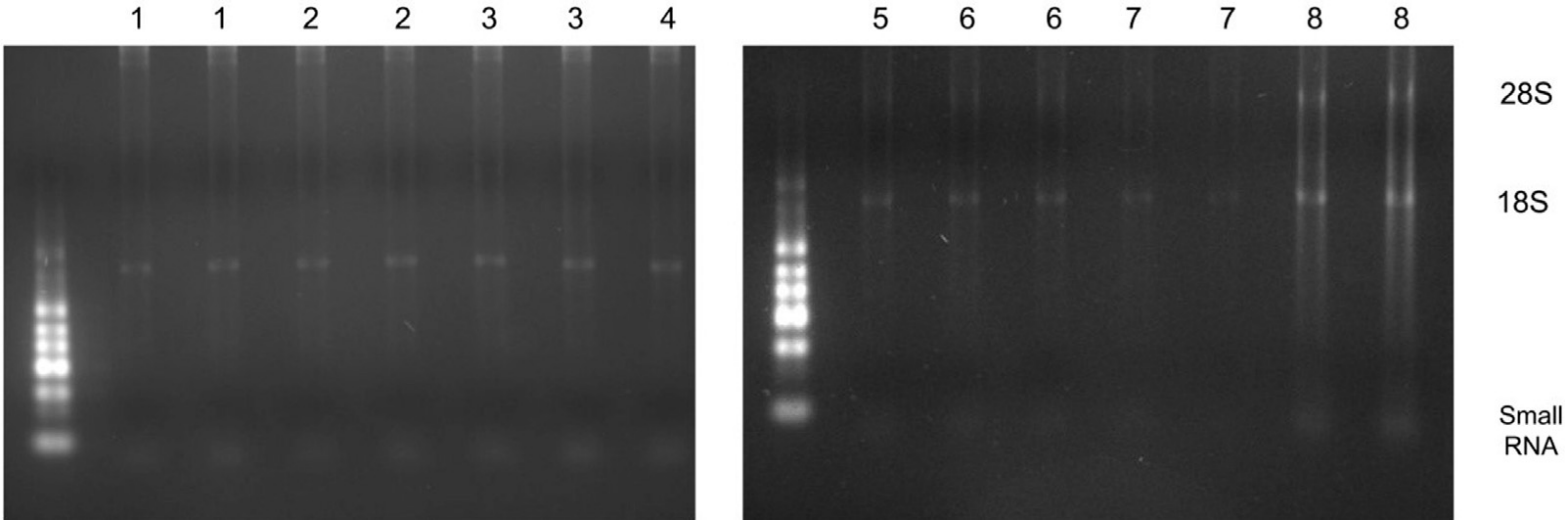
Comparison of the isolated RNA from the different groups using low range RNA ladder in a native 2% agarose gel. The numbers above the lanes indicate each of the groups in table 2. Our standardized procedure yielded optimal RNA quality as represented by the 18S and 28S rRNA bands in group 8.

Gel electrophoresis revealed that our isolated total RNA by the optimized protocol was of acceptable quality, as evident by the presence of 18S and 28S ribosomal RNA as well as small RNA (Fig. 4).

Our results show that our isolated VAT RNA samples yielded Ct values which were significantly different when compared with the unoptimised protocol for RNU6 (*p* = 0.03) and miR181 (*p* = 0.01). Further, the unoptimised method had higher Ct values for all the genes, and more variability, as shown by the coefficient of variation (CV) percentage (Table 4).

**Table 4:** Ct values (average) for GAPDH and SMAD7, and RNU6 and miR181 in five samples (*n* = 5) comparing our standardised protocol with the unoptimised protocol.

Sample	Without optimisation				With optimisation			
	GAPDH	SMAD7	RNU6	miR181	GAPDH	SMAD7	RNU6	miR181
1	23.21	25.70	20.34	26.12	21.33	23.87	18.61	24.40
2	23.26	23.70	22.97	30.01	21.27	23.62	21.19	27.17
3	22.15	25.15	23.08	29.05	19.33	22.43	20.97	25.09
4	20.62	24.17	21.88	28.26	20.69	24.17	19.19	24.90
5	24.62	23.75	24.52	29.90	22.43	25.38	21.34	26.82
CV(%)	6.5	4.8	6.9	5.5	5.3	4.4	6.2	4.8
*p*-value					0.07	0.36	0.03*	0.01*

*p<0.05 (two sample *t*-test).

Comparison by two sample *t*-test revealed significant differences between the Ct values of RNU6 and miR181.

## Discussion

In the present study, a standardised protocol for VAT was developed from the existing method of manual total RNA isolation based on the different factors affecting the quantity and purity of isolated RNA. TRIzol, a cost-effective and potent medium for RNA extraction from a diverse array of samples, has been used by researchers extensively. However, high lipid content and low cell count of adipose tissue have garnered particular interest amongst them to try out many different modifications of existing protocols [7,11,13,[15], [16], [17]]. Mendez et al. [11] optimised a protocol from a variety of tissues including adipose tissue and achieved a yield of 50 μg RNA (per 100 mg tissue) and A_260/280_ of 1.8–2.0. However, their samples included tissues from other body organs of animals, and they did not mention how much their total RNA yield was from adipose tissue alone. Other studies have got comparable or better yields but with suboptimal purity [3,6,7,9,18] (Table 5). Sharma et al. [10] extracted high-quality adipose tissue RNA from various animals, but their overall yield was less. Some of these methods are kit-based and/or time-consuming. Moreover, these studies are mostly on animal tissues. Inexpensive, manual methods for RNA extraction from scanty human VAT samples are scarce. In our case, using the optimised method, we extracted approximately 21 μg of total RNA per 500 mg of a tissue sample taken, with both A_260/280_ and A_260/230_ of 2.0 and 1.8, respectively.

**Table 5:** Comparison of RNA yields for adipose tissue RNA extraction protocols by different research groups.

Type of tissue	Site	Method used	RNA yield (in μg/mg)	260/280 ratio	260/230 ratio	Authors
Human adipose tissue	Subcutaneous and omental	TRIzol- 1.5 mL for 100 mg	0.06	1.70	–	Engeli et al. 1999 [18]
Primary human adipocytes	Mammary adipose tissue	TRIzol- 1.5 mL for 1 g or 2 × 10^5^ cells	0.02	1.58	–	Janke et al. 2001 [3]
Porcine adipose tissue	Retroperitoneal	TRI Reagent and miRNeasy (combined)	0.044	2.00	1.73	Cirera 2013 [7]
Human adipose tissue	Subcutaneous: abdominal or mammary	RNeasy Lipid Tissue Kit	0.133	–	–	Hemmrich et al. 2010 [4]
Human adipose tissue	Subcutaneous, perivascular, and epicardial	TRIzol and RNeasy kit	–	2.09	1.95	Sinitsky et al. 2018 [6]
Human adipose tissue	Subcutaneous adipose tissue	MagNA Pure Compact RNA Isolation kit	0.034	1.74	–	Lacinova et al. 2008 [9]
Animal adipose tissue	Subcutaneous adipose tissue	SDS, mercapto-ethanol and guanidium chloride extraction buffer	0.011–0.052	–	–	Sharma et al. 2017 [10]
Porcine tissues	Liver, muscle, hypophysis, adipose tissue, intestinal mucosa	TRIzol- 1 mL per 100 mg tissue powder	0.50	1.8–2.0	–	Mendez et al. 2011 [11]
Rat adipose tissue	Epididymal and perirenal	Guanidium thiocyanate extraction buffer- 1 mL per 1.5 g tissue	0.07	2.0–2.2	–	Tavangar et al. 1990 [13]
Human adipose tissue	Abdominal visceral	TRIzol- 250μL per 500 mg of tissue	0.042	1.98	1.84	Current study

Total RNA yields are normalized to μg/mg for each study, wherever data was available.

In case multiple methods were employed, the method with the best overall outcomes have been mentioned.

We showed that an increasing tissue amount increases the RNA yield significantly without compromising the purity. Also, decreasing the TRIzol volume, adding the step for removal of the fat layer, and the additional ethanol washing step have a profound effect on the improvement of the 260/230 ratio, which is in agreement with the existing literature that says residual phenol, fat, and salt contaminations are hindrances in the way of achieving optimal RNA purity. The addition of the fat layer removed step also significantly increased the RNA yield (ng/mg of VAT). When we combined the varying tissue amounts with specific TRIzol volumes for a defined tissue sample, we observed that using 500 mg of tissue with a 1:2 TRIzol-to-tissue ratio gives an RNA yield of around 0.042 μg/mg with a 260/280 ratio of 1.98 and 260/230 ratio of 1.84. Hence, for our standardised method, we kept 500 mg VAT, a 1:2 TRIzol-to-tissue ratio, the step for fat removal, and the additional ethanol wash for good results. In case 500 mg tissue is not available, 400 mg tissue also gives comparable results in terms of yield and ratio.

The RT-PCR which were run for housekeeping genes (GAPDH and RNU6), an mRNA (smad7), and a microRNA (miR181b) showed consistent Ct values with lesser variability per sample in all samples for each gene, indicating that this modified protocol is fit and superior to the standard extraction procedure for downstream methods for both coding and non-coding RNA.

However, a critical factor in this study was the between-patient variation which could influence the results, but measures were taken to keep that variation to a minimum. Using lower quantities of TRIzol results in a lower volume of upper aqueous phase, which makes it more prone for lesser yield and DNA contamination. Nevertheless, we have taken the utmost care during the phase separation step, and in our optimized protocol, the results (Table 2) show that using less TRIzol not only increases the RNA quality in terms of ratio, but also does not affect the RNA yield as less TRIzol had more yield across all starting quantities of tissue. The difference between groups was further found to be statistically significant (*p*<0.001). However, in a few groups, the group sizes are small, and sample sizes are variable between groups, which are shortcomings of this study. Any inefficiency of the ethanol washing step was countered by adding an extra washing step.

## Conclusion

This modified extraction protocol is currently being used successfully in our laboratory in downstream applications like RT-PCR with desired outcomes. Thus, we conclude that manual total RNA extraction by the phenol-chloroform method using TRIzol can be optimised by removing the extra fat layer, optimising the ratio of TRIzol to VAT, and adding an extra ethanol wash, all of which improve the quality and purity of extracted RNA.

## Data analysis

Data were analysed in R programming platform (version 3.5.3) using RStudio, and Orange toolkit [19], [20], [21]. We tested the variables for normality by graphical representation (histogram and Q-Q plot) and Shapiro-Wilk significance test. The 260/280 and 260/230 ratio were presented as median (quartiles Q1-Q3). We further tested the differences between groups. The comparisons for non-parametric variables were carried out using the Wilcoxon Rank-Sum test (for two groups) or Kruskal-Wallis test (more than two groups), and for parametric data, *t*-test was used. Post-hoc analysis for differences between groups was done using pairwise comparisons by Wilcoxon rank-sum test. Spearman's Rho test was used for the correlation between variables. For all analytical purposes, *p*-values less than 0.05 were considered to be statistically significant.

## Additional chloroform wash

**Fig. 5:**
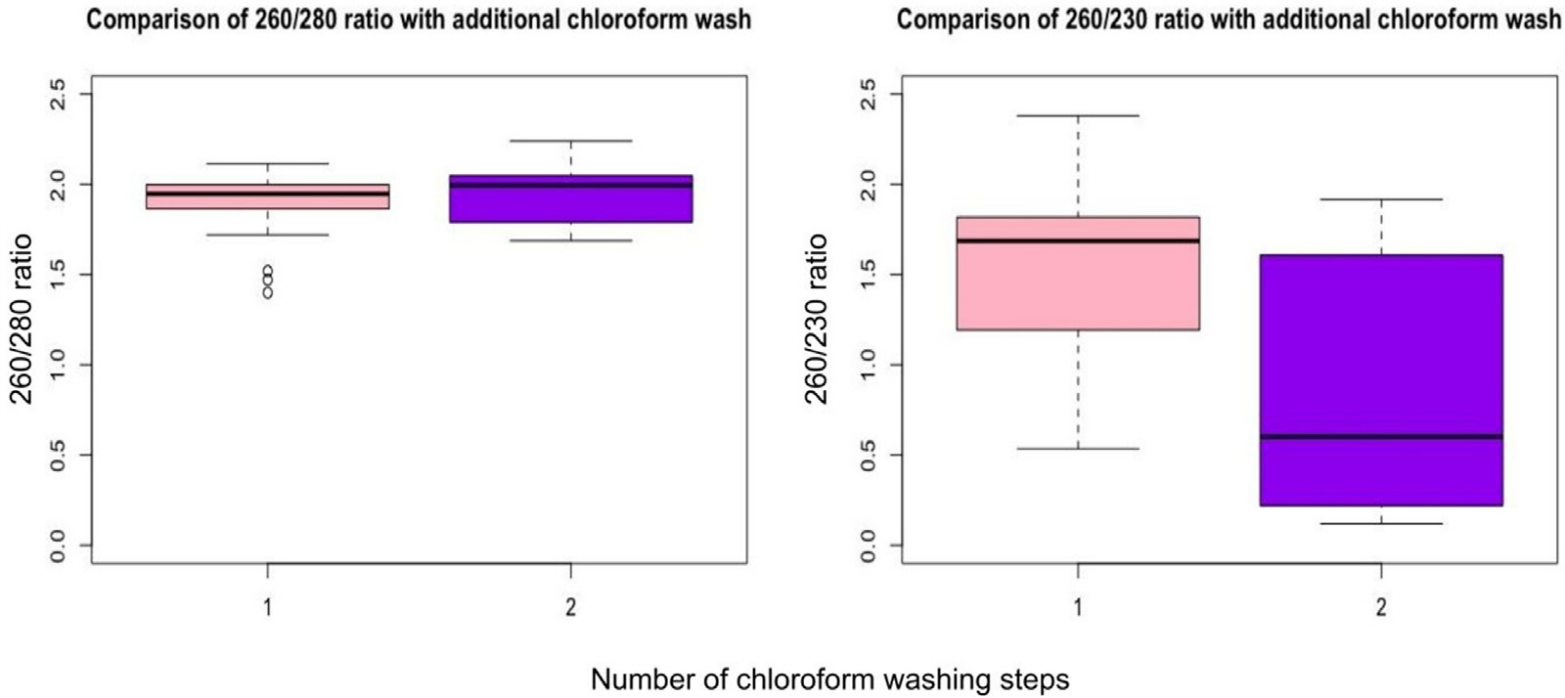
Effect of an additional chloroform wash after phase separation. Code 1 = single chloroform wash, 2 = double chloroform wash. Boxplots showing that 260/280 ratio underwent no significant change (Two sample Wilcoxon rank sum test, p = 0.18). 260/230 ratio shows significant decline with the second wash (p = 0.003). * p<0.05 (Two sample Wilcoxon rank sum test with continuity correction).

One modification that we anticipated to improve the RNA purity was not fruitful. A 2018 study [14] showed that while purifying RNA from ventricular myocytes of 1-to-2 day old Sprague Dawley rats, an extra step of chloroform wash significantly improves the RNA quality, purity, and accuracy of the quantification compared to the conventional protocol. Accordingly, we tried to see whether an extra chloroform wash also improves the RNA extracted from human VAT. There was a non-significant increase in the median for the 260/280 ratio (*p* = 0.18), but a significant decline was noticed for the 260/230 ratio (median [Q1-Q3] 0.60 [0.22–1.61] compared to 1.69 [1.19–1.82]; *p* = 0.003, Fig. 5). Hence, we decided not to include an additional chloroform wash in our standardised protocol.

## Declaration of Competing Interest

The authors declare that they have no known competing financial interests or personal relationships that could have appeared to influence the work reported in this paper.

## Appendix A. Supplementary material and additional information

Our protocol is a modified version of the manufacturer's protocol provided with the TRIzol reagent. For validation with RT-qPCR, we followed the miScript SYBR Green PCR System Handbook from QIAGEN.

Links: i) http://himedialabs.com/TD/MB601.pdf ii) https://www.qiagen.com/us/resources/download.aspx?id=0948c3fd-c643-4daa-a777-11425991ba3e&lang=en iii) https://www.qiagen.com/us/resources/download.aspx?id=7954ef25-3a39-4b0a-a27e-42689dbb4f5f&lang=en iv) *Riboruler Low Range RNA Ladder*. https://assets.thermofisher.com/TFS-Assets/LSG/manuals/MAN0013098_RiboRuler_LowRange_RNA_Ladder_UG.pdf. [for RNA agarose gel electrophoresis]
